# Disease burden among refugees in camps on mainland Greece, 2016–2017: a retrospective cross-sectional study

**DOI:** 10.1186/s12889-023-16472-3

**Published:** 2023-09-04

**Authors:** Sarah Elizabeth Scales, Jee Won Park, Rebecca Nixon, Debarati Guha-Sapir, Jennifer A. Horney

**Affiliations:** 1 Epidemiology Program, University of Delaware, Suite 614 Tower at STAR 100 Discovery Boulevard Newark, Newark, DE 19713 USA; 2 Department of Geography and Spatial Sciences, University of Delaware, 225 Pearson Hall 125 Academy Street Newark, Newark, DE 19716 USA; 3 Division of International Health, Johns Hopkins University Bloomberg School of Public Health, Baltimore, MD USA

**Keywords:** Displacement, Disaster epidemiology, Humanitarian emergency, International health

## Abstract

**Background:**

Despite the importance of baseline health data for evidence-informed decision-making, these data are rarely available for displaced populations. At the height of the European refugee crisis, most of those seeking asylum in Europe were from regions with high prevalences of communicable and non-communicable diseases. To create an epidemiologic profile for refugees in camps on mainland Greece, this study assessed the prevalence of 11 communicable and non-communicable diseases among refugees utilizing Médecins du Monde (MdM) in-camp clinics.

**Methods:**

The proportional morbidity of selected diseases among individuals utilizing MdM services were determined from data collected at refugee camp clinics on mainland Greece from April 2016 - July 2017. Overall and age-specific proportional morbidities were reported. Differences in disease burden among refugees from the largest sending countries - Afghanistan and Syria - were compared using proportional morbidity ratios and 95% confidence intervals. Patterns in results were compared with disease burden estimates in sending countries and with findings from comparable settings.

**Results:**

Respiratory tract infections (RTIs) were the most prevalent outcome. Among RTIs, upper RTIs were most common, with a proportional morbidity of nearly 40%; throughout the study period, over 46% of children under 18 years had at least one upper RTI consultation. Musculoskeletal conditions (3.64%), were the most prevalent non-communicable outcome, followed by hypertension (2.21%) and asthma (1.28%). Afghans were 31.68% more likely than Syrians to have a consultation for at least one condition (PR: 1.32; 95% CI: 1.25, 1.39). The proportional morbidity of RTIs was comparable to sending countries, but there was a comparatively lower burden of other conditions among refugees than literature estimates from sending countries.

**Conclusion:**

Refugees utilizing MdM clinics in camps had higher burdens of communicable diseases - predominantly RTIs - relative to non-communicable diseases. Non-communicable disease burdens were comparatively lower than reported prevalences from in-country populations. These findings can be attributed to a range of considerations including differences in demographic profiles between sending countries and refugee populations and missed opportunities for utilizing clinical care. Further investment is needed to capture the health profiles of displaced populations to support evidence-informed decision-making processes in humanitarian emergency responses.

**Supplementary Information:**

The online version contains supplementary material available at 10.1186/s12889-023-16472-3.

## Background

 From 2014 to 2015, more than 13 million people in the Middle East and North Africa were displaced due to conflict, with an additional 163,000 displaced due to disasters [1]. Without civil and social support structures, internally displaced persons are often forced to cross international borders in search of protections external to the government of their country of origin [2]. As of December 2015, individuals from the World Health Organization’s (WHO) Eastern Mediterranean Region (EMRO) made up over half of the total number of refugees globally, with nearly 28% and 18% fleeing Syria and Afghanistan, respectively [3]. EMRO has high prevalences of, and associated mortality due to, non-communicable diseases (NCDs), with cardiovascular, respiratory, and metabolic disease and cancers collectively accounting for more than 65% of deaths within the region [4]. Simultaneously, EMRO has a high burden of communicable and infectious diseases, partially driven by high endemicity and low immunization rates [5, 6].

The scale of displacement throughout EMRO has placed a significant burden not only on the region’s countries and people but also on neighboring communities. Greece is a gateway to the European Union (EU), and a large number of forcibly displaced persons from countries in the Middle East, Central Asia, the Horn of Africa, and North Africa - most of whom are part of EMRO - make treacherous journeys across the Mediterranean to claim asylum in the EU. For the purposes of this study, asylum seekers, refugees, and migrants will collectively be referred to as refugees. This is reflective of common practice in for discussing the situation in Greece specifically and Europe more broadly, although asylum seekers, refugees, and migrants have distinct definitions and legal classifications [7]. In 2015, record numbers of refugees entered Greece via unauthorized sea crossings, thousands of whom were unaccompanied minors [8, 9].

Without a cogent asylum process, the EU as a whole was not equipped to handle the increased levels of displacement in the Eastern Mediterranean in 2015. The 2015 EU-Türkïye deal incentivized the Turkish government to improve the status of refugees within their country and deter irregular entries into the EU (e.g., sea crossings) [10]. To control movement of refugees and asylees already in the EU, a hard border was set-up, blocking entry into the Schengen Zone (i.e., passport-free zone) of the EU accessed via the Balkan Route, a footpath commonly used to travel from Greece to Western Europe through the Balkan states [11]. These steps greatly limited the mobility of refugees, more or less confining them to camps within Greece while Greece was experiencing its own significant economic and political challenges [12]. To support Greece’s shift from a predominantly “transit country” to a “receiving country,” the EU’s Directorate for European Civil Protection and Humanitarian Aid Operations activated Médecins du Monde (MdM) - also called Doctors of the World - teams to run clinics in refugee camps on mainland Greece [13]. Since 1980, MdM has deployed to more than 30 countries to improve access to healthcare in complex and humanitarian settings [14].

The 2015 refugee crisis - which brought the realities of global displacement to the doorstep of the West - reignited discussions of displacement and health, prompting a reckoning of not only the changing dimensions of humanitarian need but also appropriate and effective ways to ensure commensurate response [15, 16]. Climate change, man-made and natural hazards, and conflict reinforce one another, creating a vicious loop of cascading and compounding disasters and humanitarian emergencies [17, 18]. While there is not a decisive position on the role of climate change on displacement [19, 20], displacement due to the complex and interrelated effects of co-occurring climate-related hazards and geopolitical instability is well documented [21, 22]. As global geopolitical stability becomes more precarious and climate change precipitates more frequent and severe disasters, the status of and response to refugees and displaced persons will become even more paramount .

Quantifying the health care needs of, and conditions experienced within, displaced populations is vital for evidence-informed humanitarian response, planning, and preparedness aimed at reducing negative health outcomes among both refugee and host populations [23]. Acknowledging the increasing complexities of humanitarian response, we undertook this study to assess the disease burden of 11 communicable (e.g., infectious diseases that can be transmitted between people, such as tuberculosis) and non-communicable (e.g., diseases or conditions that are not transmissible, such as hypertension) conditions - selected based on literature review, data availability, and subject-matter expertise - among refugees utilizing MdM clinics in mainland refugee camps from April 2016 - July 2017.

## Methods

### Study description and population

A retrospective, cross-sectional analysis of background data was conducted to address the objectives of this paper: to describe the burden of common communicable and non-communicable diseases among refugees utilizing MdM clinics in refugee camps on mainland Greece and to compare the prevalences of these conditions among those from the largest sending countries - Afghanistan and Syria.

Clinical line-list data from MdM clinics operating across 21 refugee camps on mainland Greece were used to describe the burden of common communicable and non-communicable diseases among refugees utilizing services at these clinics. Due to proximity and data recording practices, two camps - Koutsochero I and Koutsochero II - were combined for the purposes of this study, leaving 20 camps. Data were obtained from MdM and the Centre for Research on the Epidemiology of Disasters, Association pour l’Etude Epidemiologique des Désastres (CRED-ASED).

Refugees entering Europe are typically adolescents, with more males than females, compared to refugees in other parts of the world [24, 25]; these demographic features are reflected in the MdM dataset (Table 1). Further, we assume that individuals within camps had equal access to in-camp MdM clinics, so data are assumed to be a random sample of the camp populations and a fair representation of displaced persons in EMRO.

**Table 1:** Inclusion and exclusion criteria used to define cases of conditions of interest based on diagnoses, diagnosing notes, and treatment/prescribing notes recorded by MdM clinicians for consults in clinics across 20 refugee camps on mainland Greece, 2016–2017

	Inclusions	Exclusions
Asthma	Any diagnosed asthma or history of asthma	Unspecified breathing difficulty
Hepatitis B	Diagnosis notes indicating Hepatitis B	N/a
Herpes	Diagnosis notes indicating herpes, genital and oral	N/a
Hypertension / high blood pressure	Diagnoses of high blood pressure or hypertension; blood pressure checks with prescribing notes for anti-hypertensives	Pregnant women;
		Blood pressure checks without readings, additional notes, or anti-hypertensives
Insulin dependent diabetes mellitus	Unspecified diabetes with prescribed insulin without glucophage; IDDM or Type I Diabetes	Unspecified diabetes with no prescribing or diagnosing notes; unspecified diabetes with only glucophage prescribing notes
Lower-respiratory tract infections	Any diagnosed infection of the lower respiratory tract, including confirmed and suspected pneumonia, bronchitis, and bronchiolitis	Non-communicable lower respiratory tract conditions
Musculoskeletal disorders and diseases	Any musculoskeletal consultation	N/a
Non-insulin dependent diabetes mellitus	Unspecified diabetes with prescribed glucophage; NIDDM or Type II Diabetes	Unspecified diabetes with no prescribing or diagnosing notes; unspecified diabetes with only insulin prescribing notes
Tonsilitis	Any diagnosed tonsilitis or scarlet fever	Unspecified sore throat
Tuberculosis	Diagnoses of confirmed or suspected tuberculosis	Negative Mantoux; Mantoux for school or employment without results
Upper-respiratory tract infections	Any diagnosed infection of the upper respiratory tract, including common cold, scarlet fever and tonsilitis/amigdalitis, communicable sore throat, sinusitis, communicable rhinitis	Non-communicable upper respiratory tract conditions

### Measures

For the purposes of this analysis, gender and sex are used interchangeably and treated as a binary variable (male/female) in line with the United Nations’ Office for Disaster Risk Reduction’s Sendai Framework Monitor Sex, Age, and Disability Disaggregated Data (SADDD) reporting standards [26]. To have more granular information, age was disaggregated into 4-categories (0-17y; 18-39y; 40-59y; 60+) rather than the 3-category classification used by the SADDD.

Binary case definitions (yes/no) were developed for the 11 outcomes of interest: hypertension/high blood pressure, non-insulin dependent diabetes mellitus (NIDDM), insulin-dependent diabetes mellitus (IDDM), herpes, hepatitis B, lower-respiratory tract infections (LRTI), upper-respiratory tract infections (URTI), asthma, musculoskeletal disorders and diseases, tuberculosis (TB), and tonsilitis. Conditions were chosen based on literature review and data availability; further, they represent high population-level morbidity and mortality. Diagnoses, in conjunction with diagnosing and treatment/prescribing notes were used to determine cases, as detailed in Table 1. Due to lack of standardized data collection and recording processes from MdM data, case definitions were developed based on standard case definitions.

### Analysis

Overall and age-specific proportional morbidities were reported for each outcome of interest. Proportional morbidity - or the prevalence of outcomes of interest among the 7865 individuals with MdM consultations - is defined by the Hellenic Ministry of Health’s Centre for Disease Control and Prevention Department of Epidemiological Surveillance and Intervention as “consultations for a given syndrome/health condition as a proportion of total consultations (for all causes).” Accordingly, proportional morbidities were calculated as cases from the total study population, and age-specific proportional morbidities were calculated as cases from the study population within the age category. For comparisons of disease proportional morbidities between Afghan and Syrian refugees, hepatitis B was excluded from comparisons due to small sample size. Chi-squared test was used to compare demographic characteristics between Afghan and Syrian refugees. No weights were applied to proportional morbidity calculations. Data were cleaned and analyzed win SAS Studio using PROC SURVEYFREQ (Cary, NC).

## Results

There was a total study population of 7856 individuals across the 20 camps. Nearly 45% (*n* = 3508) individuals had clinical consultations for at least one condition of interest. More than 46% of all consultations were for children under the age of 18 (*n* = 3625), and 51.29% of children (*n* = 1873) had a consultation for at least one condition of interest. Demographic characteristics are presented in Table 1. The median age for all consultations was 19 years (IQR = 23) with ages ranging from under 1 year to 85 years of age. Nearly 62% of consultations were from four camps: Elliniko (23.41%), Malakasa (14.37%), Raidestos (12.4%), and Koutsochero I and II (10.90%). Afghans were the majority of the camp populations for Elliniko and Malakasa at 96.96% and 96.19%, respectively. In Koutsochero I and II (83.43%) and Raidestos (89.50%), the majority of the population was Syrian. Other sending countries for all camps included Algeria, Egypt, Eritrea, Iran, Iraq and Kurdistan, Kuwait, Lebanon, Libya, Morocco, the Occupied Palestinian Territories, Pakistan, and Somalia. Males (51.57%) had more recorded consultations than females (48.43%). More than 10% of the total number of individuals with consultations were for Afghan males under the age of 18. The demographic characteristics of individuals with MdM consultations are summarized in Table 2. Age-specific and overall proportional morbidities and 95% CIs are presented in Table 1.

**Table 2:** Demographic characteristics of refugees utilizing MdM clinics in refugee camps on mainland Greece from April 2016 - July 2017

Country of origin	n (%)
Afghanistan	3213 (40.85%)
Algeria	15 (0.19%)
Egypt	9 (0.11%)
Eritrea	1 (0.01%)
Iran	71 (0.90%)
Iraq	586 (7.45%)
Kuwait	13 (0.17%)
Lebanon	6 (0.08%)
Libya	8 (0.10%)
Morocco	8 (0.10%)
Pakistan	67 (0.85%)
Palestine	24 (0.31%)
Somalia	6 (0.08%)
Syria	3838 (48.80%)
**Sex**	**n (%)**
Female	3809 (48.43%)
Male	4056 (51.57%)
**Camp**	**n (%)**
Agia Eleni	63 (0.80%)
Doliana	139 (1.77%)
Drama	143 (1.82%)
Elliniko	1841 (23.41%)
Faneromeni/Lakkas	284 (3.61%)
Filipiada	399 (5.07%)
Grevena Hotels	50 (0.64%)
Hotel Aetopetra/ Drama	77 (0.98%)
Hotel Refanidis/Kavala	23 (0.29%)
Katsikas	614 (7.81%)
Kavala	319 (4.06%)
Kipselochori	74 (0.94%)
Konista	160 (2.03%)
Koutsochero I and II	857 (10.90%)
Malakasa	1130 (14.37%)
Raidestos	1010 (12.84%)
Schisto	168 (2.14%)
Trikala	360 (4.58%)
Tsepelevo	45 (0.57%)
Volos	109 (1.39%)
**Age**	**Median (Q1-Q3)**
19.00 years	(8.00–31.00 years)

**Table 3:** Age-specific and overall prevalences and 95% CIs for conditions of interest by age group and for all refugees with consults at MdM clinics across refugee camps on mainland Greece, 2016–2017

Condition	N, %, (95% CI)				
	Age Categories				
	0y-17	18y-39	40y-59	60+	Overall
Asthma	45 1.23% (0.87–1.59)	36 1.13% (0.76–1.50)	19 2.2% (1.2–3.1)	1 0.68% (0.00–2.01)	101 1.28% (1.04–1.53)
Hypertension	3 0.08% (0.0 0- 0.18)	28 0.88% (0.55–1.20)	94 10.64% (8.61–12.68)	49 33.11% (25.44–40.77)	174 2.21% (1.89–2.54)
Hepatitis B	0 - -	4 0.13% (0.00–0.25)	0 - -	1 0.68% (0.00–2.01)	5 0.06% (0.01–0.12)
Herpes	20 0.55% (0.31–0.79)	22 0.69% (0.40–0.98)	6 0.68% (0.14–1.22)	0 - -	48 0.61% (0.44–0.78)
IDDM	2 0.05% (0.00–0.13)	4 0.13% (0.00–0.25)	9 1.02% (0.36–1.68)	2 1.35% (0.00–3.23)	17 0.22% (0.11–0.32)
LRTI	247 6.76% (5.95–7.57)	117 3.67% (3.02–4.33)	53 6.00% (4.43–7.57)	15 10.14% (5.22–15.05)	432 5.49% (4.99–6.00)
Musculoskeletal	92 2.52% (2.01–3.03)	130 4.09% (3.40–4.77)	48 5.44% (3.93–6.93)	16 10.81% (5.75–15.87)	286 3.64% (3.22–4.05)
NIDDM	1 0.03% (0.00–0.08)	11 0.35% (0.14–0.55)	68 7.70% (5.94–9.46)	19 12.84% (7.39–18.29)	99 1.26% (1.01–1.50)
Tonsilitis	489 13.39% (12.28–14.49)	349 10.97% (9.88–12.05)	73 8.27% (6.45–10.09)	11 7.43% (3.15–11.71)	922 11.72% (11.01–12.43)
Tuberculosis	18 0.49% (0.27–0.72)	10 0.31% (0.12–0.51)	8 0.91% (0.28–1.53)	2 1.35% (0.00–3.23)	38 0.48% (0.33–0.64)
URTI	1681 46.03% (44.41–47.65)	940 29.54% (27.96–31.13)	224 25.37% (22.49–28.24)	45 30.41% (22.91–37.90)	2890 36.75% (35.67–37.81)

### Infectious and communicable diseases

The proportional morbidities of herpes (prev: 0.61%; 95% CI: 0.44–0.78%), hepatitis B (prev: 0.06%; 95% CI: 0.01–0.12%), and TB (prev: 0.48%; 95% CI: 0.33–0.64%) as identified through clinical consultations were less than 1%. Children under 18 accounted for the highest number of confirmed or suspected TB cases (*n* = 18), but the age-specific proportional morbidity was highest among adults 60 and older (prev: 1.35%; 95% CI: 0.00–3.23%).

Throughout the study period, the proportional morbidity of URTIs was 36.8% (95% CI: 35.7–37.8%), with children 0–17 years and adults 60 years and older having the highest age-specific prevalences, 46.0% (95% CI: 44.4–47.6%) and 30.4% (95% CI: 22.9–37.9%), respectively. Children younger than 18 accounted for 21.4% of all URTI consultations (95% CI: 20.5–22.3%). The proportional morbidity of tonsilitis - a type of URTI - was 11.7% (95% CI: 11.0–12.4%) and decreased across age categories. Over 13% of children with MdM consultations had at least one diagnosis of tonsilitis or scarlet fever (95% CI: 12.3–14.5%). The proportional morbidity of LRTIs was 5.5% (95% CI: 5.0–6.0%). Children under 18 and adults 60 and older had the highest age-specific proportional morbidity of 6.7% (95% CI: 5.9–7.6%) and 10.1% (95% CI: 5.2–15.1%), respectively. Children accounted for 57.2% of all LRTI cases. When further categorizing age, the proportional morbidity of LRTIs in older adults was driven by individuals 70–79 years of age, with 20.8% having at least one consultation for an LRTI (data not shown).

### Non-communicable diseases

The proportional morbidity of NIDDM was 1.3% (95% CI: 1.0–1.5%), increasing across age categories. Nearly 13% of individuals 60 and older had consultations with diagnoses or prescriptions for NIDDM (95% CI: 7.4–18.3%). Similarly, IDDM proportional morbidity increased across age categories, with a low population morbidity of less than 1% (prev: 0.2%; 95% CI: 0.1–0.3%). Hypertension was most common among adults over the age of 40 years, with the age-specific proportional morbidity of 10.7% for those aged 40–59 years (95% CI: 8.6–12.7%) and 33.1% for those 60 and older (95%CI: 25.4–40.8%). Because the population utilizing MdM camps was appreciably younger, the population proportional morbidity for the study period was much lower, at 2.2% (95% CI: 1.9–2.5%).

The proportional morbidity of musculoskeletal consultations was 3.6% (95% CI: 3.2–4.1%), with 286 individuals receiving care for injuries, wounds, pain, and other conditions. The age-specific proportional morbidity increased by age group, with the proportion of musculoskeletal conditions the highest among adults 60 and older (prev: 10.8%; 95% CI: 5.7–15.9%). The proportional morbidity of asthma was 1.3% (95% CI: 1.0–1.5%), with adults 40–59 years of age representing the highest proportion of asthma consultations (prev: 2.2%; 95% CI: 1.2–3.1%).

### Syrians and Afghans

Syria and Afghanistan were the two highest sending countries, accounting for 48.80% and 40.85% of individuals with MdM consultations, respectively. There were more men (53.00%) with consultations than women (47.00%) among Afghans; but there were slightly more women (50.63%) with consultations than men (49.37%) among Syrians. The mean age of Afghans (mean = 21.18 ±15.33; median = 20, IQR = 22) and Syrians (mean = 20.87±15.61; median = 18, IQR = 24) with MdM consultations was not statistically significantly different (p-value = 0.2799, F-value = 1.04). The median ages and interquartile ranges (IQR) and sex breakdowns of cases for Afghans and Syrians are summarized in Supplementary Tables 1 and 2.

Country-specific proportional morbidity and ratios comparing Afghans and Syrians are summarized in Table 1. LRTIs were more common among Afghans, who were 99.61% more likely than Syrians to have an LRTI (PR: 2.00; 95% CI: 1.64–2.43). The median age for Syrian cases was 8.5 years (IQR = 30), 2 years younger than the median age for Afghans. Nearly 61% of Afghan LRTI cases were male, compared to 53.29% of Syrian LRTI cases. Afghans were 48.31% more likely to have a URTI than Syrians (PR: 1.48; 95% CI: 1.40–1.58). Among Afghans, males were 54.58% of URTIs, and the median age of cases was 14 years (IQR = 23). The sex distribution among Syrians was more equal, with 51.68% of cases among males. The median age of URTIs among Syrians was 11 years (IQR = 23). Tonsilitis was three times higher among Afghans than Syrians (PR: 3.10; 95% CI: 2.69–3.57). The median age of Syrians cases (12 years; IQR = 21) was younger than for Afghans (17 years; IQR = 23). Nearly 50% of Afghans utilizing MdM clinics were diagnosed with at least one URTI, LRTI, or asthma during the study period.

**Table 4:** Age-specific and overall prevalences and 95% CIs for conditions of interest for Afghans and Syrians with consults at MdM clinics across refugee camps on mainland Greece, 2016–2017; prevalence ratios comparing prevalence of a given condition

Condition	N, %, (95% CI)										PR (95% CI)
	Afghans					Syrians					
	0y-17	18y-39	40y-59	60+	Overall	0y-17	18y-39	40y-59	60+	Overall	
Asthma	9	17	6	1	33	25	16	13	0	54	0.73
	0.63%	1.22%	1.79%	1.49%	1.03%	1.35%	1.09%	2.89%	-	1.41%	(0.47–1.12)
	(0.22–1.05)	(0.64–1.80)	(0.36–3.22)	(0.00–4.47)	(0.68–1.38)	(0.82–1.87)	(0.56–1.63)	(1.34–4.44)	-	(1.03–1.78)	
Hepatitis B	0	2	0	1	3	0	1	0	0	1	3.58
	-	0.14	-	1.49%	0.09%	-	0.07%	-	-	0.03%	(0.37–34.43)
	-	(0.00–0.34)	-	(0.00–4.47)	(0.00–0.20)	-	(0.00–0.20)	-	-	(0.00–0.08)	
Herpes	2	7	2	0	11	15	15	3	0	33	0.40
	0.14%	0.50%	0.60%	-	0.34%	0.81%	1.02%	0.67	-	0.86%	(0.20–0.79)
	(0.00–0.34)	(0.13–0.88)	(0.00–1.43)	-	(0.14–0.54)	(0.40–1.22)	(0.51–1.54)	(0.00–1.42)	-	(0.57–1.15)	
Hypertension	2	10	22	14	48	1	13	66	28	108	0.53
	0.14%	0.72%	6.57%	20.90%	1.49%	0.05%	0.89%	14.67%	42.42%	2.81%	(0.38–0.74)
	(0.00–0.34)	(0.27–1.16)	(3.90–9.23)	(10.90–30.89)	(1.07–1.91)	(0.00–0.16)	(0.41–1.37)	(11.39–17.95)	(30.18–54.67)	(2.29–3.34)	
IDDM	2	2	2	1	7	0	1	6	1	8	1.05
	0.14%	0.14%	0.60%	1.49%	0.22%	-	0.07%	1.33%	1.51%	0.21%	(0.38–2.88)
	(0.00–0.34)	(0.00–0.34)	(0.00–1.43)	(0.00–4.47)	(0.06–0.38)	-	(0.00–0.20)	(0.27–2.40)	(0.00–4.54)	(0.06–0.35)	
LRTI	139	75	31	9	254	95	31	20	6	152	2.00
	9.78%	5.40%	9.25%	13.43%	7.91%	5.12%	2.12%	4.44%	9.09%	3.96%	(1.64–2.43)
	(8.24–11.33)	(4.21–6.58)	(6.13–12.37)	(5.05–21.81)	(6.97–8.84)	(4.11–6.12)	(1.38–2.85)	(2.53–6.36)	(1.97–16.21)	(3.34–4.58)	
Musculoskeletal	26	33	16	10	85	56	83	26	5	170	0.60
	1.83%	2.37%	4.78%	14.93%	2.65%	3.02%	5.67%	5.78%	7.58%	4.43%	(0.46–0.77)
	(1.13–2.53)	(1.57–3.18)	(2.48–7.07)	(6.17–23.68)	(2.09–3.20)	(2.24–3.79)	(4.48–6.85)	(3.61–7.94)	(1.02–14.13)	(3.78–5.08)	
NIDDM	1	3	16	10	30	0	4	43	7	54	0.66
	0.07%	0.22%	4.78%	14.93%	0.93%	-	0.27%	9.56%	10.61%	1.41%	(0.43–1.03)
	(0.00–0.21)	(0.00–0.46)	(2.48–7.07)	(6.17–23.68)	(0.60–1.27)	-	(0.01–0.54)	(6.83–12.28)	(2.98–18.23)	(1.03–1.78)	
TB	16	9	4	2	31	1	0	4	0	5	7.41
	1.13%	0.65%	1.19%	2.99%	0.96%	0.05	-	0.89%	-	0.12%	(2.88–19.02)
	(0.58–1.68)	(0.23–1.07)	(0.02–2.36)	(0.00–7.17)	(0.63–1.30)	(0.00–0.16)	-	(0.02–1.76)	-	(0.02–0.24)	
Tonsilitis	317	244	54	8	623	144	78	15	3	240	3.10
	22.31%	17.55%	16.12%	11.94%	19.39%	7.75%	5.32%	3.33%	4.55%	6.25%	(2.69–3.57)
	(20.14–24.48)	(15.55–19.56)	(12.16–20.08)	(3.97–19.91)	(18.02–20.76)	(6.54–8.97)	(4.17–6.48)	(1.67–5.00)	(0.00–9.71)	(5.49–7.02)	
URTI	824	519	106	26	1475	734	339	100	15	1188	1.48
	57.99%	37.34%	31.64%	38.81%	45.91%	39.53%	23.14%	22.22%	22.73%	30.95%	(1.40–1.58)
	(55.42–60.56)	(34.79–39.88)	(26.64–36.65)	(26.82–50.78)	(44.18–47.63)	(37.30–41.75)	(20.98–25.30)	(18.37–26.08)	(12.35–33.11)	(29.49–32.42)	

The proportional morbidity of hypertension (PR: 0.53, 95% CI: 0.38–0.74) and NIDDM (PR: 0.66, 95% CI: 0.43–1.03) were lower among Afghans than for Syrians. The median age of Afghan hypertension cases was 54 years (IQR = 20); cases were evenly distributed among women and men. Women were 51.85% of Syrian hypertension cases; the median age for all Syrian cases was 52 years (IQR = 17). The median age for Syrians with NIDDM was 52 years (IQR = 6); 53.70% of cases were among men. The median age for Afghans with NIDDM was 55 years (IQR = 15) with 60.0% of cases among women.

Musculoskeletal consultations were higher among Syrians than Afghans, with Afghans being 40.00% less likely to have a musculoskeletal condition than Syrians (PR: 0.60, 95% CI: 0.46–0.77). For both Syrians and Afghans, more males than females had musculoskeletal conditions. The median age of individuals with musculoskeletal consultations was 26 years (IQR = 30) and 24 years (IQR = 23) for Afghans and Syrians, respectively. Asthma was less common among Afghans than Syrians (PR = 0.73; 95%CI: 0.47–1.12). Among Afghans, 63.64% of asthma cases were male compared to half of Syrian cases. The median age of Afghan asthma cases was 28 years (IQR = 28) and 22 years (IQR = 36) for Syrians.

## Discussion

After the fall of Kabul on 15 August 2021, Rahimitabar et al. (2023), conducted a systematic review to better understand the healthcare needs of Afghans in Germany and Iran, where 45% of Afghans who resettle in Germany spend a year or more in-transit [27], noted that there are less data and fewer studies addressing health status of refugees in Germany compared to Iran [28]. This is reflective of the gap that exists as refugees move and resettle further from their countries of origin. The findings of this study contribute new insights into the health profile of refugees after making the treacherous journey across the Mediterranean, adding important context to data and studies from EMRO-neighbor countries such as Lebanon, Jordan, Iran, and Türkïye.

Because national and regional estimates of non-communicable diseases are sparse throughout EMRO and Africa WHO regions, the results of this study provide important context to the diseases experiences of populations displaced from these regions. As noted throughout grey and published literature, the burden of non-communicable diseases throughout EMRO is not adequately captured, resulting in undercounting of disease burden and higher individual- and population-level impacts due to untreated or unregulated illness [29, 30]. Underlying chronic diseases like hypertension and metabolic disorders contribute to increased morbidity and mortality from infectious and communicable diseases [31]. Accordingly, understanding proportional morbidity of non-communicable disease can not only inform program- and system-level interventions for prevention and treatment but also reduce communicable disease morbidity and mortality associated with higher risks due to underlying chronic conditions. Because of the high morbidity and mortality - both direct and indirect - associated with hypertension and diabetes, these conditions are discussed in detail below.

### Hypertension

A 2014 systematic review of hypertension prevalence, awareness, and control in Arab countries highlighted that, while these regions have high burdens of obesity, diabetes, and smoking, population-level estimates for hypertension for the regions are underexplored [30]. Using published estimates from 10 Arab countries, the same estimated the prevalence of hypertension to be roughly 30% across studies from 10 Arab countries [30]. Other in-country-of-origin studies gave estimates of 40.6% in Aleppo, Syria [32]; 28.4% for adults 25–65 years in Jalalabad City, Afghanistan [33]; and 46.2% for adults 40 and older in Kabul, Afghanistan [34]. The proportional morbidity of hypertension and high blood pressure in MdM clinics was notably lower at 2.21% (95% CI: 1.89–2.54) for the entire study population and 4.06% for those over the age of 18 years (95% CI: 3.46–4.66). Roughly 14% of individuals over the age of 40 years had consultations for hypertension, and 33.11% of individuals 60 years and older had consultations for hypertension (95% CI: 25.44–40.78).

The comparatively lower observed prevalence of hypertension between sending country and refugee populations was noted in other studies. Doocy et al. (2013), found that nearly 20% of Iraqis displaced to Jordan and Syria who participated in a cross-sectional survey were hypertensive compared to national estimates of nearly 30% for the same time period [35]. Our findings could be attributed to differences in data collection; in-country studies used household surveys, going to individuals to ascertain hypertensive status through self-report and/or biometrics. By using clinical data, we have only captured individuals who have clinical symptoms of disease and/or who have a previous diagnosis and know to seek continued care and/or medication refills. A study of healthcare access and health seeking behavior among refugees in New Zealand found that, in addition to systemic level barriers to access, individual-level perceptions, language skills, and expectations influenced an individual’s healthcare utilization [36]. However, the prevalence of hypertension among Syrian refugees in primary care settings in Türkïye, Lebanon, and Jordan was estimated at 35% from January 2011 through November 2021 [37]. This is still notably higher than overall and Syrian-specific estimates for hypertension in MdM clinics. Because refugees are generally accepted to be healthier than their peers who stay in-country, it follows that the prevalence of hypertension among MdM patients is truly lower than in-country estimates. Further, lower proportional morbidity in MdM clinics compared to primary care settings in EMRO transit countries could be attributed to the “healthy refugee” concept, with individual-level health being better among those whose journeys are spatially and temporally of longer duration [38].

### Diabetes

In a cross-sectional, population-based survey conducted in Aleppo, Syria, before the start of the Syrian Civil War, the prevalence of NIDDM was 15.6% when measured as fasting blood sugar from blood samples [39]. Extrapolating the findings, Albache et al. (2010), found that, among adults 25 and older in Aleppo, the prevalence of NIDDM and elevated fasting glucose was nearly 25%. Estimates from the second and third largest sending countries, Afghanistan and Iraq, respectively, also had in-country estimates higher than the proportional morbidity of NIDDM among MdM clinic users. In 2016, the Afghan Ministry of Public Health, in collaboration with WHO, estimated that 8.4% of Afghans were living with diabetes, predominantly non-insulin dependent [29]. A 2019 report from the Iraqi Ministry of Health estimated that diabetes affected roughly 14% of all Iraqis and accounted for 4% of all deaths [40]; the 2015 STEPS survey found that nearly 10% of Iraqi adults had undiagnosed NIDDM.

In our study, less than 1% of individuals with MdM consultation had IDDM (95% CI: 0.11–0.32%) while 1.26% of individuals had NIDDM (95% CI: 1.01–1.51%); for both conditions, proportional morbidity was highest among adults 60 and older. As observed with hypertension, proportional morbidity of NIDDM among adults 60 and older was more comparable to in-country estimates, with proportional morbidity of roughly 13% for these individuals (95% CI: 7.39 − 18.29%).

Consistent with observations at a global level, individuals with hypertension were appreciably more likely to have NIDDM than individuals without hypertension. Although the population burden of IDDM and NIDDM are not notably high, the population-level impacts and associated negative health outcomes can have persistent knock-on effects for both individuals and the health systems in receiving countries. Both study design (e.g., cross-sectional) and underlying demographic differences in refugee and in-country populations (e.g., age, sex) could contribute to differences between in-country prevalences and MdM proportional morbidity. Further, the MdM proportional morbidity of NIDDM is lower than the true population-level burden of disease; fifty-five additional diabetes cases were excluded because of insufficient information about type.

### Musculoskeletal disorders

Musculoskeletal issues, including chronic pain and wounds, are very common among displaced populations [41–44]. Among Syrian refugees in Sultanbeyli District, Türkïye [42], the prevalence of musculoskeletal impairments was 12.2%, which is higher than the observed prevalence of 4.43% (95% CI: 3.79–5.08%) among Syrians using MdM clinics. Adult Afghans in Qom and Tehran, Iran, had a high burden of musculoskeletal conditions [43]. Among recently arrived Afghans in Germany utilizing primary care services, the prevalence of musculoskeletal and/or connective tissue disorders was 9.3% [45], again higher than the observed prevalence of 2.65% (95% CI: 2.09–3.20%) among Afghans using MdM clinics.

Nissen et al. (2022), investigated the relationship between physical pain and psychosocial stressors among Syrians resettled in Norway, highlighting the potential of psychosomatic pain as the physical manifestation of mental health conditions such as post-traumatic stress disorder [46]. In the MdM data set, the prevalence of musculoskeletal consultations was 3.64% (95% CI: 3.22–4.05%), with the prevalence of these consultations increasing with age. However, mental health consults accounted for 5.33% of all MdM consultations; the age-specific prevalence was highest for those 40–59 years at 11.44%. More than 4% of adults aged 40–59 years (95% CI: 3.94–6.93%) and 10.81% of adults 60 and older (95% CI: 5.75–15.87%) had musculoskeletal disorders. It is reasonable that there are missed somatoform cases of musculoskeletal pain that were recorded as mental health consultations, which were outside of the scope of this study. Further study into the relationship between musculoskeletal disorders and mental health is needed to understand if and how these aspects of physical and mental health affect refugees in mainland Greece.

### Respiratory diseases

Exposure, malnutrition, underlying illness or conditions, and crowded housing accommodations are a few of the multifactorial causes of the high burden of respiratory infections among displaced populations [47, 48]. Refugees in camps on mainland Greece utilizing MdM clinics similarly experienced a high burden of respiratory infections. Week-to-week, syndromic surveillance reports from the Hellenic Ministry of Health showed respiratory infections with fever to have the highest proportional morbidity of the 14 routinely monitored syndromes [49]. More than 39% of all MdM consultations were for either upper or lower RTIs (95% CI: 38.09–40.25). Nearly 50% of children 17 years and younger had at least one consultation for a RTI (95% CI: 47.01–50.25), and 31% of adults 18 years and older had at least one consultation for an RTI (95% CI: 29.58–32.37). The prevalence of asthma - which can modify risks of contracting RTIs and subsequent clinical severity of disease - was 1.28% (95% CI: 1.04–1.53). The high proportional morbidity of consultations for respiratory infections for both children and adults are reflective of similar contexts. Across 20 host countries with data available through the Refugee Health Information System in 2019, upper and lower RTIs accounted for 24% of under-5 morbidity, and LRTIs accounted for nearly 6% of under-5 mortality [50]. Acute respiratory infections were the most frequent cause for clinical visits among adult refugees at reception centers in Germany [51].

The proportional morbidity of asthma in the MdM population is notably lower than survey prevalences of 12.5% in Kabuli primary school aged children [52]; 8.5% in children 5 years and older in Damascus, Syria; and 22.3% among primary-school children in Baghdad, Iraq. Asthma is likely underestimated among the MdM dataset, which presumably only captures severe or exacerbated cases necessitating clinical consultations.

The high burden of respiratory infections among refugees in camps on mainland Greece highlights the importance of implementing simple and effective disease control measures. Because RTI burden is influenced by environmental and circumstantial factors such as season, housing facilities, and population density [48]; environmental control measures to reduce crowding - especially in confined spaces - and water, sanitation, and hygiene protocols are standard practice for addressing risk of infection from both respiratory and other pathogens. Further, on-arrival, catch-up vaccinations for respiratory infections (e.g., seasonal flu, influenza B, diphtheria, pertussis, COVID-19) and contribute to improved individual and population-level risk of transmission and illness.

### Other communicable diseases

The proportional morbidity of both TB and hepatitis B were less than 1% among individuals utilizing MdM clinics (TB 95% CI: 0.33–0.64%; hepatitis B 95% CI: 0.02–0.12%). While both diseases have appreciable population-level impacts and are contagious, especially in crowded spaces and among populations in-flux, latent and asymptomatic infections for TB and hepatitis B, respectively, can make case finding through clinical data challenging. However, there are actionable steps to address both individual- and population-level risks associated with such communicable diseases.

Four countries of origin had TB cases - Afghanistan, Iraq, Morocco, and Syria. However, the burden of TB would be expected to be higher overall, with cases from additional sending countries. This could be attributable in part to the fact that MdM consultations for TB only capture symptomatic cases, and, less frequently, indolent cases caught through Mantoux testing for school attendance or employment.

The incidence of TB throughout EMRO varies greatly, with Pakistan and Afghanistan accounting for nearly 80% of the region’s cases [53]. A systematic review covering TB screenings from 1991 to 2017 found that the incidence of TB was heterogenous across both populations and time [54]. However, population burden of TB was consistently higher among refugee populations than in host communities [54, 55]. Another meta-analysis found that the heterogeneity in pooled incidence of latent and active TB was driven by differences in country of origin [56]. Assessments of TB burden in refugee and host communities, such as displaced Syrians in Lebanon and Jordan, use active case-finding and active surveillance methods [55].

However, syndromic surveillance from reception centres across Greece consistently reported relatively few cases of suspected tuberculosis, with non-significant changes in observed and expected case numbers during the same time period as our study [49]. Surveillance and screening biases could account for the comparatively lower burden of TB among refugees utilizing MdM clinics in mainland refugee camps. Regardless of the number of TB cases, linking identified cases to care, conducting drug resistance screenings, and ensuring completion of treatment regimens is needed for reducing individual risk of negative health outcomes as well as for reducing population-level health risks associated with TB.

A meta-analysis assessing prevalence of hepatitis B throughout EMRO from 2000 to 2016 estimated the prevalence of antibodies against hepatitis B to be 2.84% [57]. Lee et al. (2023), found that there is limited investigation into hepatitis B in refugee populations despite available information indicating high disease burden [58]. Hepatitis B, which has long-term disease sequalae, is a vaccine preventable disease. Although hepatitis B-containing vaccines are indicated throughout EMRO countries, coverage estimates for 1-year olds in EMRO ranged from 41% in Syria to 99% in Morocco for 2015 [59]. A 2010–2013 assessment of laboratory measures for refugee children in Greece found that few children had either vaccine- or infection-derived antibodies against hepatitis B [60]. Ensuring that all refugees have access to routine vaccination upon arrival in camps - whether through targeted vaccination campaigns or inclusion in national vaccination programs - is important for reducing the burden of vaccine-preventable diseases like hepatitis B.

### Syrians and Afghans

Despite the similarities of circumstance and need among all refugees, health-related needs are unique to specific refugee populations. The importance of considering specific needs for distinct populations groups has been noted since the 1980s. Following the 1970s and 80s refugee crisis resulting from United States military actions in Southeast Asia, Catanzaro and Moser (1982) highlighted the importance of disaggregating refugee populations rather than considering all refugees, regardless of country of origin or ethnic background, to be the same [61]. Similar findings were shared for refugee and immigrant children in Greece from 2010 to 2013 [60]. However, disaggregation by country of origin and/or ethnicity is not always the standard of practice. Our study contributes to this gap in the literature by comparing Afghan and Syrians utilizing MdM clinics. Syrian and Afghan refugees utilizing MdM clinics had appreciably different burdens of a number of conditions.

Syrians were more prone to chronic conditions like hypertension and musculoskeletal issues as well as herpes. Afghans had appreciably more consultations for upper and lower RTIs and tuberculosis although both Syrian and Afghan refugees had high burdens of RTIs. While refugees using MdM clinics from both countries were - on average - young, it is likely that more Afghan minors were unaccompanied compared to Syrian minors, adding another dimension of health risk. Of those seeking asylum in the EU in 2015, roughly half of unaccompanied minors were Afghans, predominantly boys, while Syrians made up 16% of unaccompanied minors [62]. A cross-sectional study of unaccompanied minors seeking asylum in Germany found the prevalence of infectious diseases among these children to be high, while the prevalence of non-communicable diseases was considerably lower [63].

In a qualitative study of the experiences and perspectives of Afghan and Syrian refugees resettled in Europe, Belabbas et al. (2022), demonstrated Afghans and Syrians have faced similar circumstances and challenges throughout their migration journeys [64]. However, both the spatial and temporal duration of migration journeys are different between Syrians and Afghans. For example, common transit countries for Syrians include Jordan, Lebanon, and Turkey while Pakistan, Iran, and Turkey are common transit countries for Afghans [27]. The circumstances - integration of refugees into host communities, strength of social support and health care institutions, general treatment of and feelings toward refugees - vary greatly not only between countries but also at from across host communities within the same country. These factors can influence both physical and mental health of refugees upon arrival in transit or final countries; differences in transit routes could contribute to differences in communicable and non-communicable disease burdens between Afghan and Syrian refugees.

### Limitations

As noted throughout the discussion, the lower-than-expected prevalences could be due to a number of explanations. Migration is physically and psychologically strenuous; accordingly, younger and healthier individuals are over-represented among refugee populations. This can partially explain the lower-than-expected prevalences of non-communicable diseases.

The individuals utilizing MdM clinics may not be representative of the entirety of refugees in Greek camps. The basic demographics are reflective of the demographic profile of refugees, migrants, and asylum seekers in Europe [65], supporting the assumption that individuals with consultations are a reasonably random sample of these populations living in mainland Greek camps. For instance, the slight male skew in asylum seekers and refugees in Europe is partially driven in part by the number of unaccompanied Afghan male minors [25]; the data used in this study have a similar gender skew, and more than 10% of the total number of individuals with consultations were for Afghan males under the age of 18.

In this study, proportional morbidity from camps is compared with findings from studies reporting population prevalences. While the sex-and-age distributions of individuals with MdM consultations are reflective of refugee populations, there could be bias in comparing prevalences of those with consultations to prevalences from populations at large. Most studies reporting disease prevalence among refugee populations are household surveys. Because data for our study were collected from clinical consultations, only those with conditions necessitating clinical care or maintenance were captured. While some individuals used MdM clinics for prescriptions, there were other resources for acquiring needed medications. Therefore, there are likely well-managed or sub-clinical cases of outcomes of interest that were not represented in the MdM clinical data.

Because of MdM clinic records, including diagnostic and prescribing notes, were not standardized across clinicians, cases may be missed due to differences in the collection and recording of data from different practitioners. Many of the 7865 individuals had multiple consultations for both the same and different conditions of interest. The issue of double, triple, and more visits for a condition for a single individual was handled by creating binary variables for the conditions of interest.

## Conclusions

This work shows that there are differences in burden of both communicable and non-communicable diseases for refugees from different countries of origin. There is added value to not only assessing burdens of disease among refugee populations at large but also assessing burdens of disease within and between refugees from different countries of origin. Identifying differences in disease burden between Afghan and Syrian refugees can provide meaningful insights that allow practitioners to develop community-specific, targeted, and appropriate responses. It is out of the scope of this study to explore the totality of the mechanisms which drive differences in disease experience by country of origin; collaborative research undertakings among social sciences, epidemiology, and clinical medicine researchers are needed to better understand the influences of individual- and population-level migration experiences on refugee health and well-being.

While the health profile of displaced populations is connected with disease experiences in their respective sending countries, these data show the need to create epidemiologic profiles which capture the specific health-related needs of displaced populations rather than solely relying on external estimates from ministries of health or comparable emergency settings. To better facilitate the provision of effective and equitable humanitarian aid, understanding the unique health needs of refugee populations is essential. This also has ramifications for disaster risk reduction and preparedness. As seen in the February 2023 earthquakes in Türkïye and Syria, displaced populations are often relegated to areas with high risks of disasters caused by natural hazards, in addition to the health threats posed by instability, conflict, and displacement. If the underlying health profile of a community is not known before a disaster, it is logistically impossible to measure how the disaster caused or modified the affected community’s disease experience. Further, knowing the underlying health profile would allow for both humanitarian and disaster responses to be targeted at specific needs.

### Supplementary Information

**Additional file 1.**

## Data Availability

The datasets analyzed during the current study are not publicly available at the discretion of the original data holders, but data are available from the corresponding author upon reasonable request.

## Abbreviations

CI: Confidence interval
EMRO: Eastern Mediterranean Region
EU: European Union
IDDM: Insulin-dependent diabetes mellitus
LRTI: Lower respiratory tract infection
MdM: Médecins du Monde
NIDDM: Non-insulin diabetes mellitus
PR: Prevalence ratio
Prev: Prevalence
RTI: Respiratory tract infection
TB: Tuberculosis
URTI: Upper respiratory tract infection
WHO: World Health Organization
