# Supplementary material for: Disease burden among refugees in camps on mainland Greece, 2016–2017: a retrospective cross-sectional study
Source: BMC Public Health. 2023 Sep 4;23:1715. doi: 10.1186/s12889-023-16472-3 (PMC10476303; doi:10.1186/s12889-023-16472-3)
Supplement: Supplementary file 1 — Additional file 1. [file 12889_2023_16472_MOESM1_ESM.docx]

**Supplementary Table 1.**

| **Condition** | **Median years (IQR)** | |
| --- | --- | --- |
|  | **Afghans** | **Syrians** |
| **Asthma** | 28 (28) | 21.5 (32) |
| **Hepatitis B** | 27 (43) | 35 (0) |
| **Herpes** | 28 (15) | 23 (19) |
| **Hypertension** | 54 (21.5) | 52 (17) |
| **IDDM** | 35 (21) | 52 (7.5) |
| **LRTI** | 10.5 (27) | 8.5 (30) |
| **Musculoskeletal** | 26 (30) | 24 (23) |
| **NIDDM** | 55 (16) | 52 (6) |
| **Tonsils** | 17 (23) | 12 (21) |
| **Tuberculosis** | 17 (17) | 46 (8) |
| **URTI** | 14 (23) | 11 (23) |

Median age and IQR for conditions of interest for Afghans and Syrians.

**Supplementary Table 2.**

| **Condition** | **N (%)** | | | |
| --- | --- | --- | --- | --- |
|  | **Afghans** | | **Syrians** | |
|  | **Males** | **Females** | **Males** | **Females** |
| **Asthma** | 21 (63.64) | 12 (36.36) | 27 (50.00) | 27 (50.00) |
| **Hepatitis B** | 1 (33.33) | 2 (66.67) | 1 (100.00) | 0 (0.00) |
| **Herpes** | 7 (63.64) | 4 (36.36) | 18 (54.55) | 15 (45.45) |
| **Hypertension** | 24 (50.00) | 24 (50.00) | 51 (48.15) | 56 (51.85) |
| **IDDM** | 3 (42.86) | 4 (57.14) | 3 (37.50) | 5 (62.50) |
| **LRTI** | 154 (60.62) | 100 (39.37) | 81 (53.29) | 71 (46.71) |
| **Musculoskeletal** | 50 (58.82) | 35 (41.48) | 105 (61.76) | 65 (38.24) |
| **NIDDM** | 12 (40.00) | 18 (60.00) | 29 (53.70) | 25 (46.30) |
| **Tonsils** | 361 (57.94) | 262 (42.05) | 118 (49.17) | 122 (50.83) |
| **Tuberculosis** | 21 (67.74) | 10 (32.26) | 4 (80.00) | 1 (20.00) |
| **URTI** | 805 (54.57) | 670 (45.42) | 614 (51.68) | 574 (48.32) |

Number and percentage of cases for conditions of interest for Afghans and Syrians.
